# Coevolution Analysis of HIV-1 Envelope Glycoprotein Complex

**DOI:** 10.1371/journal.pone.0143245

**Published:** 2015-11-18

**Authors:** Reda Rawi, Khalid Kunji, Abdelali Haoudi, Halima Bensmail

**Affiliations:** 1 Computational Sciences and Engineering Center, Qatar Computing Research Institute, Hamad Bin Khalifa University, Doha, Qatar; 2 Division of Genetics and Genomics, Boston Children’s Hospital, Harvard Medical School, Boston, MA, United States of America; 3 King Abdullah International Medical Research Center, King Abdulaziz Medical City, Riyadh, Saudi Arabia; Chinese Academy of Medical Sciences, CHINA

## Abstract

The HIV-1 Env spike is the main protein complex that facilitates HIV-1 entry into CD4^+^ host cells. HIV-1 entry is a multistep process that is not yet completely understood. This process involves several protein-protein interactions between HIV-1 Env and a variety of host cell receptors along with many conformational changes within the spike. HIV-1 Env developed due to high mutation rates and plasticity escape strategies from immense immune pressure and entry inhibitors. We applied a coevolution and residue-residue contact detecting method to identify coevolution patterns within HIV-1 Env protein sequences representing all group M subtypes. We identified 424 coevolving residue pairs within HIV-1 Env. The majority of predicted pairs are residue-residue contacts and are proximal in 3D structure. Furthermore, many of the detected pairs have functional implications due to contributions in either CD4 or coreceptor binding, or variable loop, gp120-gp41, and interdomain interactions. This study provides a new dimension of information in HIV research. The identified residue couplings may not only be important in assisting gp120 and gp41 coordinate structure prediction, but also in designing new and effective entry inhibitors that incorporate mutation patterns of HIV-1 Env.

## Introduction

**Fig 1:**
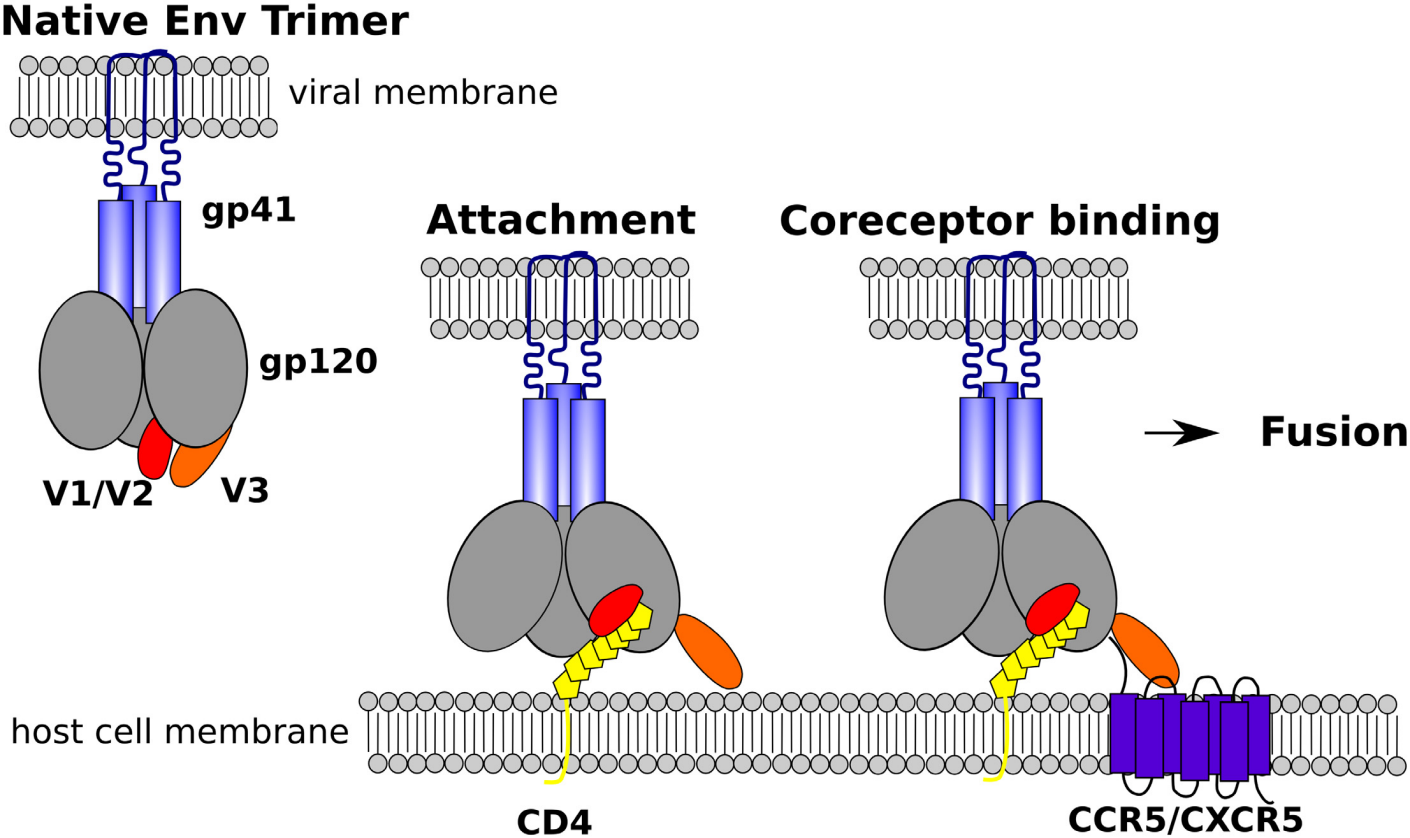
HIV cell entry. Schematic illustration of HIV-1 entry steps attachment and coreceptor binding.

Human immunodeficiency virus type 1 (HIV-1) envelope (Env) glycoprotein complex mediates binding and entry into human host cells. It is a heterodimer composed of a non-covalently bound exterior surface glycoprotein 120 (gp120) and transmembrane glycoprotein 41 (gp41) located as trimers at the surface of the viral membrane. The surface of the protein complex is highly glycosylated, enabling evasion of immune pressure. The entry process involves three main steps (see Fig 1). The attachment, initiated by the interaction of gp120 and the Cluster of Differentiation 4 Receptor (CD4), which triggers major conformational changes in gp120, including the formation of the bridging sheet (BS), spatial approach of inner (ID) and outer domain (OD) (as defined by Kwong et al. [1]) and the detachment of the variable loop 3 (V3), resulting in formation and exposure of the chemokine coreceptor binding site [1–5]. Next, the coreceptor binding, where gp120 binds in general either C-C Chemokine Receptor 5 (CCR5) or C-X-C Chemokine Receptor 4 (CXCR4), causing further conformational changes that lead to re-arrangements of the previously inaccessible gp41 into an intermediate state in which the fusion peptide of gp41 is embedded into the host cell membrane. The final step is the fusion of the viral and host cell membranes. Despite that several crystal and cryo-electron microscopy/tomography structures of gp120 in unliganded state exist [6–25] (as well as in complex with CD4, CD4 mimics, or various antibodies, and of gp41 in intermediate and post-fusion state), a comprehensive understanding of structural arrangements and communication within gp120 and gp41 domains during entry is far from complete. Interestingly, even though HIV-1 Env is target of immense immune pressure, revealed through extensive sequence diversity in the Env gene, it still maintains the protein complex structure and entry functionality. Hence, detection of coevolution of important sites in Env sequences may not only point out interesting biological interactions, but also highlight functional constraints of protein structure that could help in decrypting the complexity of function and communication during HIV entry.

The extraction of coevolution patterns out of a multiple sequence alignment (MSA) has been targeted by numerous studies during the past decades [26–31] (a recent review is provided by de Juan et al. [32]). For many years such methods required large numbers of homologous and variable protein sequences, and were not able to distinguish between real direct couplings and indirect correlations that arise from phylogenetic relationships within the sequences. Recent methodological improvements, incorporated in methods such as PSICOV [33], DCA [34, 35], plmDCA [36] or GREMLIN [37, 38] have overcome the drawbacks and demonstrated enormous accuracy in predicting real couplings and coevolution.

The majority of previous work, that studied coevolution within HIV-1 Env focused on the third variable loop (V3) [39–41], applying different sets of sequence subtypes with widely different prediction outcomes. The first coevolution study that considered the complete Env gene was performed by Travers and co-authors [42], where they included several HIV-1 group M subtypes (A,B,C,D,F,G,H,J,K) to identify coevolving pairs present among all subtypes. A recent study by Garimalla et al. [43] applied the coevolution detecting method DCA [35] on clade B HIV-1 gp120 protein sequences. Two other recent studies by Zhao et al. [44] and Li et al. [45] applied DCA and an ensemble of coevolution detecting techniques on a set of HIV-1 proteins.

In this study, we used the GREMLIN (Generative REgularized ModeLs of proteINs) approach, the most accurate method currently available for detecting coevolving residue pairs out of MSAs, and predicted 424 coevolving residue pairs within Env. The majority are real residue-residue contacts and are proximal in one of the gp120 or gp41 coordinate structures. Furthermore, we detected many coevolving pairs that have functional implications, such as CD4 or coreceptor binding, or variable loop, gp120-gp41, and interdomain interactions.

This new information should be considered in future coordinate structure predictions, but also when designing new and effective entry inhibitors to account for possible resistance mutations. To date, only two inhibitors have been approved; Maraviroc, a CCR5 antagonist that prevents the interaction between gp120 and CCR5 by blocking the transmembrane coreceptor cavity within the coreceptor, and T-20, a fusion inhibitor that prevents the fusion of the viral and host cell membranes by binding to gp41.

## Materials and Methods

### Dataset and Alignment

The input MSA was obtained from the HIV sequence database (http://www.hiv.lanl.gov/). We downloaded the filtered web alignment consisting of all group M subtype sequences including recombinants from the year 2013. The filtered web alignment represents a pre-cleaned alignment, excluding sequences with large insertions, high content of ambiguity codes, and multiple frame shifts. We subsequently applied several filtering steps. Initially we removed all sequences that contain non-standard amino acids or a gap. Next, we applied the pre-processing protocol suggested by the GREMLIN developers, which is composed of three additional steps. In the first step, we extracted all sequences from the MSA that have more than 25% gaps, followed by the removal of all columns with more than 25% gaps. The final filtering step was processed using HHfilter, a part of HHsuite (version: 2.0.15) [46], to generate a non-redundant MSA at 90% sequence identity. The final input MSA is available in (S1 File).

### Protein coordinate structures

The Protein Data Bank [47] (http://www.rcsb.org) was accessed to obtain seven HIV-1 Env crystal coordinate structures to evaluate the residue-residue contact predictions. We applied crystal structures representing gp120 in complex with CD4 and neutralising antibodies (PDB ID: 1GC1 [1], PDB ID: 2B4C [11], PDB ID: 2QAD [12]), gp120 in complex with antibody VRC01 (PDB ID: 3NGB [17]), gp120 including a gp41-interactive region (PDB ID: 3JWD [16]), stabilised HIV-1 Env in complex with antibodies PGT122 and 35O22 (PDB ID: 4TVP [48]), and the first stabilised trimeric structure of HIV-1 Env in complex with PGT122 (PDB ID: 4NCO [49]). A residue-residue contact prediction was considered true if the two coevolving amino acids are proximal in one of the seven 3D coordinate structures, in particular, if their *C*_*β*_-*C*_*β*_ (*C*_*α*_-*C*_*α*_ in the case of glycine) distance is less than 8 Ångström (Å) or their minimum atomic distance is less than 6 Å. This approach has been applied by Jones and coauthors [33].

### GREMLIN

GREMLIN [37, 38] is a method to learn a statistical model that simultaneously captures conservation and coevolution in a MSA applying a pseudo-likelihood approach. It constructs a global statistical model of the paired alignment, assigning a probability to every amino acid sequence by optimising a regularised pseudo-likelihood objective fitness function in a statistically consistent method to estimate two parameters: position-specific amino acid propensities and amino acid coupling between positions. Previous approaches estimated those two parameters using an approximate moment matching approach by inverting a generalised covariance matrix [33, 35]. These rely on a Gaussian-like approximation to the global partition function. Unlike these approaches, estimation via the pseudo-likelihood avoids this approximation relying instead on local partition functions [36, 37]. The resulting general regularised structure learning is equivalent to an optimisation problem that is efficiently solved using standard convex optimisation techniques and provides estimates for both parameters.

## Results

The first and most critical step during coevolution analysis is the construction of the protein MSA. Hence, we obtained the filtered pre-made web HIV-1 Env alignment from the HIV sequence database to ensure the quality of the alignment. We restricted the analysis to the top *L*/2 predictions (in our case 424), with *L* as the number of columns in the MSA (in our case *L* = 847). This number of top predictions has previously been applied by many research groups to benchmark their coevolution and residue-residue contact detecting methods, including the GREMLIN [38] developers. Further, Michel et al. [50] showed in their structure prediction application, applying Rosettas ab initio folding tool [51], that the consideration of *L*/2 predicted couplings as distance constraints, showed the best performances in the case of PSICOV [33] and plmDCA [36]. We identified coevolving pairs of amino acids in all gp120 and gp41 domains (see Table 1 and S1 Table). It is striking that a large number of coevolving residue pairs (in detail 54) are predicted within the first variable loop (V1), considering that the loop is composed of only 24 amino acids. In general, it was noteworthy that the variable loops in gp120 account for more than 30% of the coevolving pairs, despite that the fraction of amino acids is only around 17% of the total HIV-1 Env length. Furthermore, they identified more interdomain coevolving pairs.

**Table 1:** Count of coevolving residue pairs within and between specific HIV-1 Env regions.

	SP	C1	V1	V2	C2	V3	C3	V4	C4	V5	C5	FP	Ecto	TM	Endo
SP	29	2	0	0	0	0	0	0	0	0	0	0	0	0	0
C1		6	1	0	7	0	1	0	1	0	0	0	1	0	0
V1			54	0	0	1	0	0	0	0	0	0	0	0	0
V2				24	1	2	0	0	2	0	0	0	0	0	0
C2					34	2	10	0	4	1	4	0	1	0	0
V3						24	2	1	3	0	0	0	0	0	0
C3							34	4	0	2	0	0	0	0	0
V4								37	0	0	0	0	0	0	0
C4									8	3	0	0	0	0	0
V5										6	1	0	0	0	0
C5											2	0	2	0	0
FP												5	0	0	0
Ecto													40	0	0
TM														0	2
Endo															58

HIV-1 Env regions: signal peptide (SP), conserved regions (C1–C5), variable loops (V1–V5), fusion peptide (FP), ectodomain (Ecto), transmembrane domain (TM) and endodomain (Endo).

To evaluate the performance of the coevolution predictions we applied seven gp120 coordinate structures (see Materials and Methods). A prediction was considered true if the coevolving residues had a *C*_*β*_-*C*_*β*_ (*C*_*α*_-*C*_*α*_ in the case of glycine) distance less than 8 Å or a minimum atomic distance less than 6 Å in at least one of the seven structures. The structural analysis revealed that the majority of predicted crystallised coevolving pairs are in contact; 84% of the predictions are true positive (TP). However, we also identified long-distance coevolving residue pairs that may play important roles as interdomain, alternative conformation or binding-partner contacts.

### Predicted coevolving pairs in V3

**Fig 2:**
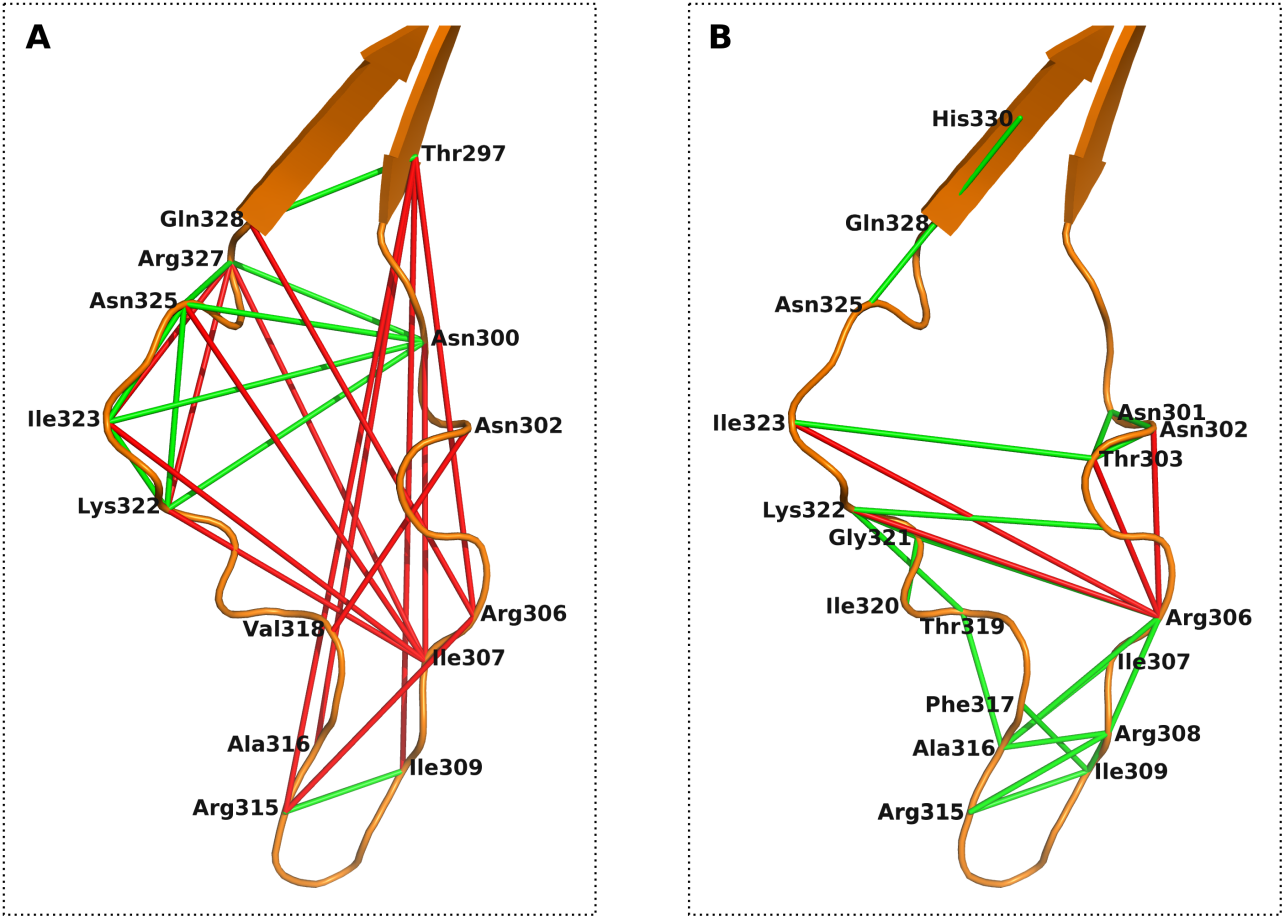
Predicted coevolving residue pairs within V3. TP predicted coevolving pairs are connected with a green dash and the FP ones are shown as red bonds. Amino acid numbering is according the HXB2 reference sequence and the V3 coordinate structure solved by Huang et al. [11] is applied for visualisation. (A) Travers and co-authors [42] identified 24 coevolving pairs of which the majority is FP. (B) Coevolution predictions made by GREMLIN identified almost exclusively TP.

V3, a highly sequence- and structure-variable loop within gp120, is of essential functional, immunological and structural importance during the entry of HIV into human host cells. Previous coevolution studies in HIV-1 Env mainly focused on V3 and identified several coevolving amino acid pairs [39–42]. In our study, we identified 24 coevolving pairs within V3 (see Fig 2B and S2 Table), of which only four are false positive (FP). We mapped the predicted residue pairs on the HIV-1 gp120 coordinate structure solved by Huang et al. [11] and highlighted them as connected coloured bonds (TP shown in green and FP in red) in Fig 2B (all figures were generated using PyMOL software [52]). To compare our results with previous work [42], we mapped their predictions on the same coordinate structure shown in Fig 2A and detected that only nine out of 24 coevolution predictions are TP. Interestingly, all FP predicted contacts in our study involve residues that are either coreceptor binding (Thr303, Arg306, Ile323) or coreceptor specific sites (Asn302, Thr303, Arg306, Asp322, Ile323), according to Korber and Gnanakaran [53] (residue numbering is according to the HXB2 reference sequence, Uniprot [54] ID: P04578). The predicted coevolution between these residues is most likely mediated through their interaction with one of the chemokine receptors, either CCR5 or CXCR4, and, hence, a typical example for an interaction partner mediated coevolution.

**Fig 3:**
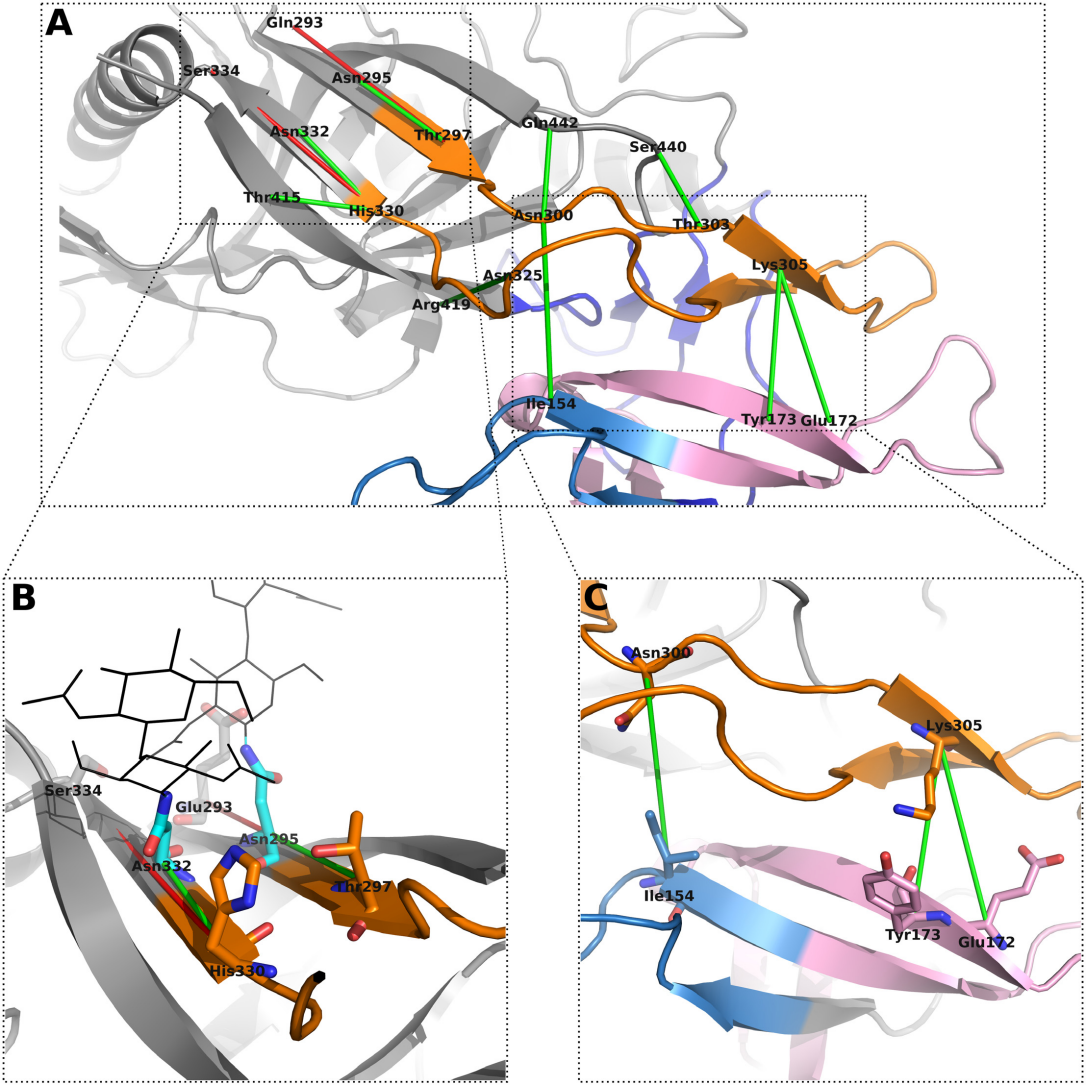
Predicted coevolving pairs between amino acids located in V3 and other structural regions of HIV-1 Env. Gp120 is shown in cartoon representation, with V1 coloured in blue, V2 in pink and V3 in orange. (A) All inter V3 coevolving pairs are highlighted with green (TP) or red (FP) coloured dashes. (B) Coevolving amino acid pairs Glu293-Asn295, Glu293-Thr297, His330-Asn332 and His330-Ser334 (shown in sticks representation) are mediated by N-linked glycans (shown as black lines). (C) Predicted contacts between amino acids located in V1V2 and V3. The involved amino acids are highlighted as coloured sticks.

Next to coevolving pairs within V3, we also identified eleven coevolving residues located in V3 and other structural regions in the HIV-1 Env glycoprotein complex (see Fig 3A and S3 Table), with two of the residue pairs predicted as FP, in particular the pairs Glu293-Thr297 and His330-Ser334. The interaction between these two coevolving pairs is also mediated through a binding partner, N-linked glycans (see Fig 3B). Among the eleven predictions we identified three coevolving residue pairs located in V1 and the second variable loop (V2), and V3 respectively, in particular Ile154-Asn300, Glu172-Lys305 and Tyr173-Lys305 (see Fig 3C). The three missing coevolving pairs (out of the eleven) are Thr303-Ser440, Asn325-Arg419 and His330-Thr415. The first two pairs are binding mediated coevolving pairs. The third pair, His330-Thr415, might represent an interesting coevolution pair, with His330 reported as coreceptor binding [53] and Thr415, located at the end of variable loop 4 (V4), adjacent to critical residues that maintain gp160 processing and maturation [55].

The plentitude and composition of intra- and inter-coevolution of V3 residues reflects the functional and structural importance of V3 during the entry into host cells. Further, this coevolution suggests extensive communication across the whole protein complex.

### Predicted coevolving pairs in V1V2

**Fig 4:**
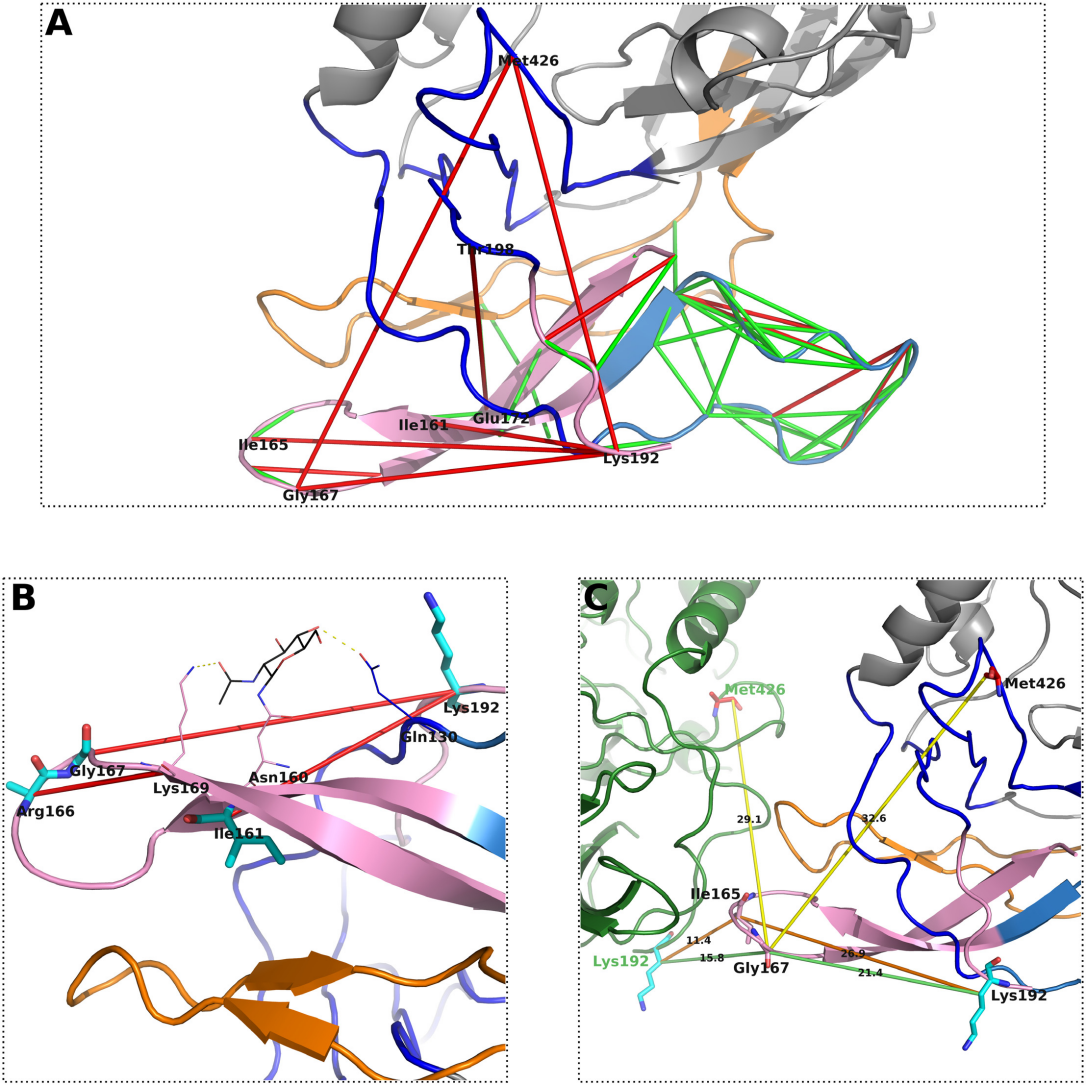
Coevolving pairs between amino acids in V1V2 and other structural HIV-1 Env domains. V1, V2 and V3 are shown in skyblue, pink and orange coloured cartoon illustration. (A) 59 predicted and in [49] crystallised coevolving residue pairs; with TP illustrated as green and FP as red dashes. (B) Two long-distance coevolving amino acids are quite likely mediated by a N-linked glycan. The involved amino acids are shown in stick representation. (C) Three long-distance residue pairs (Ile165-Lys192, Gly167-Lys192, and Gly167-Met426) are presumably inter gp120 contacts. The intra- and inter-gp120 distances are shown as coloured (orange,light green and yellow) bonds. The inter-gp120 distances are in all cases smaller than the intra-gp120 ones.

As previously mentioned, it has been reported that the V1V2 domain is important in shielding the coreceptor binding site and in conformational control of gp120 structure [22]. In our study, we identified 85 coevolving residue pairs that include at least one residue from the V1V2 region (see S1 Table). Out of the 59 intra-domain pairs, 47 are TP and 12 FP. Interestingly, we identified only coevolving pairs between residues either within V1 or V2, but no residue coevolution between the two loops. In Fig 4A we highlighted the 59 predicted residue pairs as green and red bonds. V1 is coloured skyblue and V2 pink, while V3 is indicated in the background in orange and the BS is shown in dark blue. The FP predicted residue pairs within V1 have minimum atomic distances between 6.29 Å for amino acid pair Glu150-Ile154 and 11.42 Å for Met149-Ile154. We presume that the FP predicted pairs may be TP in other conformations, since previous studies reported that the V1V2 region is in motion upon interaction with CD4 and the coreceptors. In fact, the recently published work by Munro et al. [56] showed that the unliganded HIV-1 Env is intrinsically dynamic, by transitioning between three distinct conformations. Hence, the predicted residue pairs may be TP in one of the three characterised conformations. Furthermore, we identified N-linked glycan mediated long-distance coevolving pairs within V2, similar to V3. The involved pairs are Ile161-Lys192 and Gly167-Lys192, with Ile161 adjacent to a glycan binding asparagine amino acid, Asn160, which was recently shown to be among the essential N-linked glycosylation sites that interfere in the interaction with monoclonal antibodies such as 2G12 [57].

The coevolving pair Arg166-Lys169 may also have an effect on the glycan binding by contributing with Lys169 as direct binding partner of the glycan (see Fig 4B). The coevolving pair Gly167-Lys192 might also be an inter-gp120 contact within the Env trimeric complex (see Fig 4C), with a smaller atomic distance to the neighbouring gp120 than the intra-gp120. The same applies for two other pairs, Ile165-Lys192 and Gly167-Met426. In particular, the two long-distance coevolving pairs, Gly167-Lys192 and Gly167-Met426, might represent interesting communication sites between functionally important regions, such as Met426 as a CD4 binding residue located adjacent to the Phe43 cavity, and Gly167 as adjacent to coreceptor specific and N-linked glycan binding site.

### Predicted coevolving pairs including CD4 binding residues

**Fig 5:**
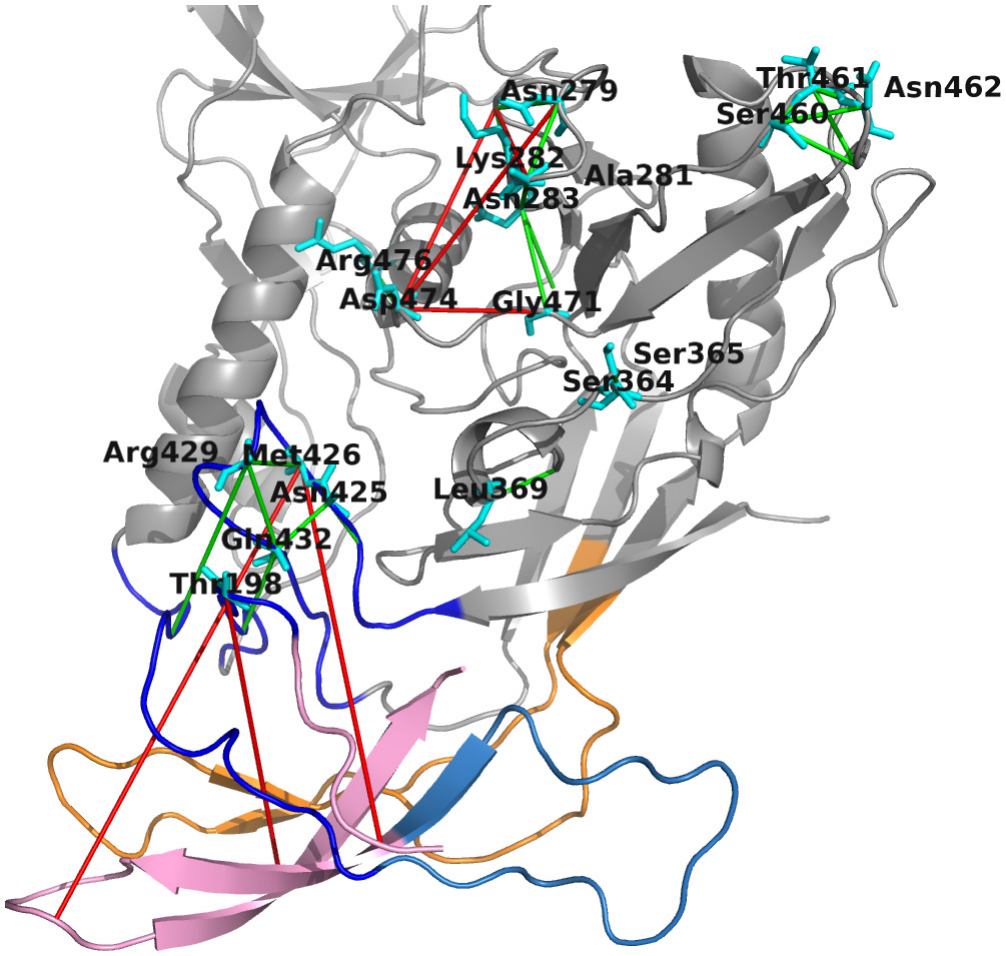
CD4 coevolution network. The coevolution network is composed of residues that directly bind CD4, highlighted as cyan coloured sticks and labeled in black. Detected coevolving pairs are shown as green (TP) or red bonds (FP).

HIV entry into host immune cells is initiated by the interaction of gp120 and CD4, which triggers conformational change in the Env protein complex. We investigated coevolving residue pairs, including residues that directly bind CD4 and residues that coevolve, but are not direct binding partners of CD4 (see Fig 5). Among the 27 coevolving pairs, only seven are FP. Four of these FP coevolving pairs are present in a subnetwork located above the Phe43 cavity of gp120, at the nexus of the bridging sheet (BS), inner domain (ID) and outer domain (OD). The remaining three long-distance coevolving pairs are located in the BS and V2 and might play key roles in inter-gp120 domain interaction, intra-gp120 communication connecting important CD4 binding residues located at the BS with residues adjacent to N-linked glycan binding and coreceptor specific sites, or different conformational arrangements of gp120 since it is well documented in previous work that conformational change is triggered following CD4 binding. Furthermore, it is worth mentioning that the residues within this CD4 coevolution network are located in different regions of gp120, in particular the BS, OD, V2, V4, as well as V5.

### Inter gp120-gp41 coevolving residue pairs

We identified four coevolving pairs between residues located in gp120 and gp41 (see Table 2). Two of the pairs are proximal in the coordinate structure solved by Pancera et al. [48], although separated by more than 100 amino acids in the sequence. The coevolving pair Val84-Ala578, although predicted as FP, involves two important residues, with Val84 adjacent to Val85, which has been previously reported as gp41 interacting [16], and Ala578, recently showed [58], that when mutated, influences the sensitivity of HI viruses to fusion/entry inhibitors T-20 and C34, by reduced anti-HIV-1 activity and decreased *α*-helicity of the gp41 N-terminal heptad-repeat.

**Table 2:** Predicted coevolving pairs between residues located in gp120 and gp41.

	Pos i		Pos j		GREMLIN score	TP
17	502	C5	607	gp41	0.72	1
184	500	C5	619	gp41	0.33	1
317	84	C1	578	gp41	0.16	0
416	238	C2	630	gp41	0.23	0

**Fig 6:**
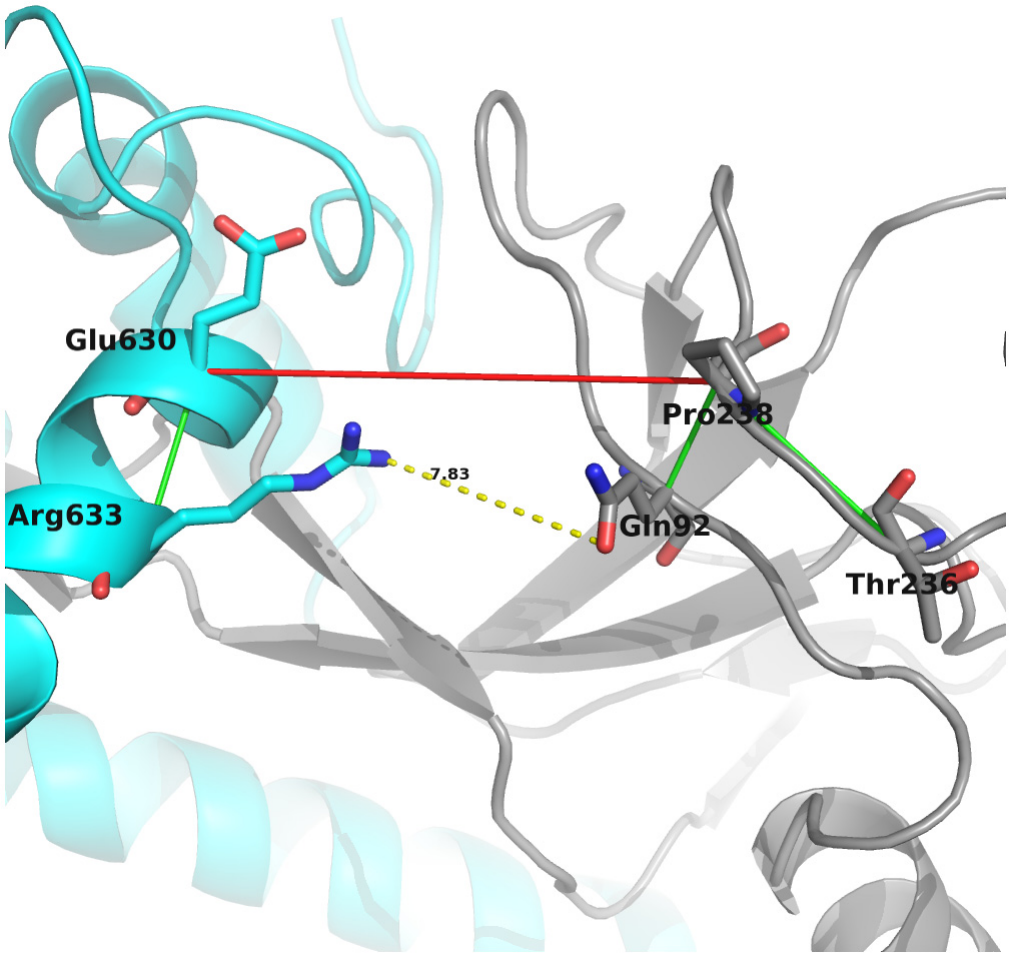
Coevolution network of the inter gp120-gp41 coevolving pair Pro238-Glu630. This pair may affect the gp120-gp41 interaction, although their are not proximal, through their intra-domain coevolving residue partners.

The last pair within this subset, Pro238-Glu630, might be coevolving within a subnetwork that affects gp120-gp41 interaction. Pro238 is further coevolving with residues Gln92 and Thr236, and Glu630 with Arg633 (see Fig 6). The coevolving partners of Pro238 (Gln92) and Glu630 (Arg633) have a minimum atomic distance of 7.83 Å. Also, Gln92 and Pro238 are reported to be gp41 interface contacts.

### Intra gp41 coevolving residue pairs

Among our 424 predictions, we identified 105 coevolving residue pairs within gp41 (see S1 Table). However, due to the lack of a complete gp41 coordinate structure that comprises all functional regions, we were not able to judge all coevolving pairs according to structural proximity. Nevertheless, we applied the 3D-structure solved by Pancera et al. [48] and evaluated the coevolving pairs whose residues are crystallised, by splitting the predicted intra-gp41 pairs into two subsets. In the first subset we included residue pairs adjacent in sequence with a maximum distance of five. The majority of predicted coevolving pairs, 76, are adjacent in sequence and within the first subset. The residues of 20 out of the 76 pairs are structurally solved and all of them are TP. We assume that the remaining pairs are also proximal in structure and TP due to the adjacency in sequence. More than half of the residue pairs, 44 out of 76, are located in the endodomain of gp41 with 7 pairs coevolving between residues located in the highly immunogenic region, known as the Kennedy epitope.

The second subset is composed of 29 coevolving pairs, with five residue pairs crystallised in the coordinate structure solved by Pancera and co-authors [48]. Only one out of five pairs is TP. However, the remaining four residue pairs are also structurally proximal with minimum distances between 8.22 Å and 11.8 Å. Out of 29 coevolving pairs, 16 are predicted between residues located in the endodomain of gp41, which is C-terminal to the viral membrane-spanning domain.

## Discussion

In this study, we successfully predicted coevolving pairs of residues within Env across all HIV-1 group M subtypes. We also identified residues of high biological interest, whose evolution is under functional, structural and interactional constraints. Previous coevolution studies within Env, mainly focussed on V3 and detected subtype specific coevolution [39–41]. Travers et al. [42] were the first that considered the complete Env gene in their analysis applying a coevolution detecting method based on substitution correlations [59]. Recent methodological improvements that establish a global statistical model from the MSA and infer direct contacts that disentangle directly from indirectly coupled positions, are more suitable in this context. Therefore, we applied the GREMLIN approach, the most accurate method currently available, for detecting coevolving residue pairs out of MSAs.

Within the top *L*/2 predicted pairs (424 pairs), we detected that the variable loops in gp120 account for more than 30% of the coevolving pairs. Such a concentration of coevolving pairs within the variable loops is not surprising, considering that coevolution detecting methods require variations at the sequence level. Travers and co-authors [42] identified more coevolving pairs in the conserved rather than in the variable regions of gp120. Remarkably, 54 coevolving pairs have been observed within V1, a small loop composed of only 24 amino acids. Despite this, V1 and V2 are highly sequence flexible due to immense immune pressure, but still maintain functionality in shielding the coreceptor binding site from antibodies and are involved in glycosylation.

HIV-1 V3 plays a crucial role in coreceptor binding and is the main determinant of coreceptor usage. Previous studies suggested a two-fold interaction of V3 with the coreceptor, pinpointing the interaction of the tip with the coreceptor’s binding pocket and the base with the coreceptor’s N-terminus. We predicted coevolving pairs within and between residues in V3 and other Env domains. The identified intra-V3 pairs turned out to be almost exclusively TP, applying a structural performance criterion that evaluates structural proximity. Applying the same performance criteria on Travers et al. [42] intra-V3 predictions, we identified that the majority are FP (see Fig 2). Nevertheless, we also observed four FP within our intra-V3 subset that may hint at a binding-partner mediated coevolution between the residues, since the involved amino acids are known to be either coreceptor binding (Thr303, Arg306, Ile323) or coreceptor specific sites (Asn302, Thr303, Arg306, Asp322, Ile323). The FP-predicted coevolving pairs might also present a critical intra-V3 communication, since it has been shown that Arg306, among other residues located at the tip of V3, is an important amino acid involved in the interaction of V3 with the chemokine receptor binding pocket, whereas its coevolving residue partners (Asn302, Thr303, Asp322, Ile323) are required in the interaction with the N-terminal part of the receptors [12, 60]. Beyond that, we identified coevolving pairs between residues located in V3 and other Env domains, amongst others a binding-partner mediated coevolution, the N-glycan mediated coevolution between amino acid residue pairs Glu293-Thr297 and His330-Ser334, and a coevolution between residues in V1V2 and V3 (see Fig 3). The important interaction between V1V2 and V3 has already been reported and emphasised by several groups [61–67], describing it as a mechanism of HIV to shield the coreceptor binding site, located around the stem of V3, from antibodies. However, in most of the previous studies they inferred the interactions between V1V2 and V3 from low-resolution electron-microscopy structures. In this study, we pinpoint the interacting amino acid pairs, which are in particular Ile154-Asn300, Glu172-Lys305 and Tyr173-Lys305. The first coevolving pair Ile154-Asn300 is a critical V1V2—V3 communication, since this is the only coevolving residue pair including a residue located in V1 and another Env domain. In addition, Asn300, located next to a critical glycan binding site and involved in coreceptor binding, has the coevolving residue partner Gln442 (see Fig 3A), which also performs interaction with the coreceptor. The other two remaining coevolving pairs, Glu172-Lys305 and Tyr173-Lys305, include Lys305 located in V3, which according to Schnur et al. [60] is also involved in coreceptor binding. One of the two coevolving partners is Tyr173, which was recently highlighted as one of two tyrosines (sulfatated form) in V2 that mediate and stabilise intramolecular interaction between V2 and V3 by mimicking the sulfated tyrosines in chemokine receptor CCR5 and antibodies such as 412d [65]. The second coevolving partner of Lys305 is the neighbouring Glu172. This residue has other coevolving partners (see S1 Table), such as the residue Tyr198, located in the BS. Tyr198 is an interesting residue within the BS, because it is not only adjacent to a glycan binding site, but also a CD4 contact residue and coreceptor specific [17, 53].

Furthermore, we have emphasised many coevolving pairs that are located in other Env regions, such as V1, V2 or the ID and OD, and that are also binding-partner mediated, either by N-glycans or CD4. We illustrated a CD4 network including residues that directly bind CD4 and their coevolving residue partners (see Fig 5). Within this network we identified coevolving pairs that might be involved in intra- or inter-protein communication, especially the pairs Asp167-Met426 and Arg192-Met426. Previous studies [1, 3] showed that upon CD4 binding major conformational re-arrangements take place, including the detachment of V3. The identified coevolving residues might be part of functionally important locations that maintain overall protein functionality effecting conformation and communication within the HIV-1 Env trimer.

In addition, we identified many coevolving residues within gp41. Most of the detected pairs are adjacent in sequence and, hence, most likely proximal in structure. Due to the lack of complete coordinate structures of gp41 in different states during HIV entry, we were not able to assign biological meanings to all pairs. Nevertheless, Travers et al. [42] identified coevolving pairs that support the model suggested by Hollier and Dimmock [68] that the C-terminal part of gp41 consists of 3 membrane-spanning domains and 2 ectodomains, a major and a minor. However, evidence against the suggested model has been presented by Postler and co-authors [69]. Their experiments point to the conventional model composed of one membrane spanning domain without any extracellular loops. Within our identified endodomain set of coevolving residues, we were not able to identify coevolving residues that specifically support one of the two models.

Despite that we assigned biological explanations to the majority of identified coevolving pairs, some of the residue couplings might be due to intra- or inter-protein communication to conserve Env functionality during the process of entry into host cells. However, some might just be real FP, although the GREMLIN approach proved to be very sensitive, especially when considering only the top *L*/2 predictions.

This coevolution study adds a new dimension of information to consider in HIV research. The most interesting coevolving residue pairs, for instance those located in the variable loops, may be evaluated for their importance in future mutagenesis studies. Newly-designed entry inhibitors or antibodies, including attachment inhibitors targeting gp120, coreceptor antagonists, or fusion inhibitors targeting gp41 should account for coevolution information to anticipate possible resistance mutations that may emerge within coevolving networks of the targeted residues.

## Supporting Information

S1 File HIV-1 Env MSA. Filtered web alignment of all HIV-1 Env group M subtype protein sequences from the year 2013 in FASTA format.
(FASTA)

S1 Table Coevolving residue pairs in HIV-1 Env. (PDF)

S2 Table Coevolving residues within V3. (PDF)

S3 Table Coevolving pairs between residues in V3 and other structural regions in the HIV-1 Env. (PDF)
