# Correction: Coevolution Analysis of HIV-1 Envelope Glycoprotein Complex

**DOI:** 10.1371/journal.pone.0145974

**Published:** 2015-12-23

**Authors:** Reda Rawi, Khalid Kunji, Abdelali Haoudi, Halima Bensmail

There are errors in affiliation 2 and affiliation 3 for author Abdelali Haoudi. Affiliation 2 should be: King Abdullah International Medical Research Center, King Saud Bin Abdulaziz University for Health Sciences, National Guards Health Affairs, Riyadh, KSA. Affiliation 3 should be: Department of Genetics and Genomics, Boston Children’s Hospital, Harvard Medical School, USA.
