# Supplementary material for: Coevolution Analysis of HIV-1 Envelope Glycoprotein Complex
Source: PLoS One. 2015 Nov 18;10(11):e0143245. doi: 10.1371/journal.pone.0143245 (PMC4651434; doi:10.1371/journal.pone.0143245)
Supplement: S1 Table — (PDF) [file pone.0143245.s002.pdf]

Supplementary information for:

Coevolution Analysis of HIV-1 Envelope Glycoprotein Complex

Reda Rawi[1,*], Khalid Kunji[1], Abdelali Haoudi[2,3], Halima Bensmail[1]

1 Computational Sciences and Engineering Center, Qatar Computing Research Institute, Doha, Qatar
2 Division of Genetics and Genomics, Boston Children's Hospital, Harvard Medical School, Boston, MA, USA
3 King Abdullah International Medical Research Center, King Abdulaziz Medical City, Riyadh, Saudi Arabia

∗ Corresponding author
E-mail: rrawi@qf.org.qa (RR)

Table S1: Coevolving residue pairs in HIV-1 Env.

	i (HXB2)	i (Domain)	j (HXB2)	j (Domain)	GREMLIN	TP
1	398	V4	400	V4	1.19	1
2	411	V4	413	V4	1.15	1
3	474	C5	476	C5	1.14	1
4	289	C2	291	C2	1.11	1
5	230	C2	232	C2	1.08	1
6	135	V1	137	V1	1.06	1
7	396	V4	398	V4	1.02	1
8	332	C3	334	C3	0.98	1
9	137	V1	139	V1	0.97	1
10	402	V4	404	V4	0.96	
11	362	C3	363	C3	0.93	1
12	356	C3	357	C3	0.85	1
13	461	V5	463	V5	0.80	1
14	283	C2	453	C4	0.80	1
15	295	C2	297	V3	0.78	1
16	620	Ectodomain	624	Ectodomain	0.73	
17	502	C5	607	Ectodomain	0.72	
18	465	V5	467	V5	0.70	1
19	777	Endodomain	778	Endodomain	0.69	
20	293	C2	337	C3	0.67	1
21	187	V2	189	V2	0.66	
22	145	V1	147	V1	0.66	
23	632	Ectodomain	636	Ectodomain	0.65	
24	279	C2	281	C2	0.65	1
25	356	C3	358	C3	0.64	1
26	398	V4	399	V4	0.64	1
27	446	C4	448	C4	0.63	1
28	136	V1	138	V1	0.63	1
29	234	C2	236	C2	0.62	1
30	334	C3	336	C3	0.61	1
31	138	V1	140	V1	0.61	1

Continued on next page

Table S1 – continued from previous page

	i (HXB2)	i (Domain)	j (HXB2)	j (Domain)	GREMLIN	TP
32	140	V1	142	V1	0.60	
33	410	V4	412	V4	0.60	1
34	360	C3	465	V5	0.60	1
35	397	V4	399	V4	0.59	1
36	30	SP	32	SP	0.59	
37	139	V1	141	V1	0.58	
38	13	SP	20	SP	0.58	
39	397	V4	398	V4	0.58	1
40	337	C3	339	C3	0.57	1
41	399	V4	400	V4	0.57	1
42	400	V4	402	V4	0.56	
43	362	C3	364	C3	0.56	1
44	290	C2	340	C3	0.55	1
45	97	C1	275	C2	0.55	1
46	144	V1	146	V1	0.55	
47	321	V3	322	V3	0.55	1
48	231	C2	268	C2	0.55	1
49	747	Endodomain	758	Endodomain	0.54	
50	188	V2	190	V2	0.54	1
51	134	V1	136	V1	0.54	1
52	399	V4	401	V4	0.54	
53	133	V1	135	V1	0.53	1
54	138	V1	139	V1	0.53	1
55	170	V2	172	V2	0.53	1
56	400	V4	401	V4	0.53	
57	393	V4	395	V4	0.53	1
58	348	C3	351	C3	0.52	1
59	403	V4	404	V4	0.51	1
60	139	V1	140	V1	0.51	1
61	275	C2	279	C2	0.51	1
62	306	V3	308	V3	0.51	1
63	147	V1	149	V1	0.50	
64	187	V2	188	V2	0.50	
65	137	V1	138	V1	0.50	1
66	85	C1	229	C2	0.50	1
67	275	C2	278	C2	0.50	0
68	401	V4	403	V4	0.49	
69	231	C2	267	C2	0.49	1
70	519	FP	520	FP	0.48	
71	290	C2	337	C3	0.48	1
72	774	Endodomain	775	Endodomain	0.48	
73	401	V4	402	V4	0.48	
74	229	C2	230	C2	0.46	1
75	412	V4	413	V4	0.46	1
76	85	C1	87	C1	0.46	1
77	411	V4	412	V4	0.46	1
78	219	C2	225	C2	0.45	1
79	325	V3	419	C4	0.45	1
80	293	C2	295	C2	0.45	1
81	134	V1	135	V1	0.45	1
82	778	Endodomain	779	Endodomain	0.45	

Continued on next page

Table S1 – continued from previous page

	i (HXB2)	i (Domain)	j (HXB2)	j (Domain)	GREMLIN	TP
83	513	FP	514	FP	0.45	
84	135	V1	136	V1	0.45	1
85	136	V1	137	V1	0.44	1
86	65	C1	208	C2	0.44	1
87	152	V1	154	V1	0.44	1
88	618	Ectodomain	620	Ectodomain	0.43	
89	755	Endodomain	758	Endodomain	0.43	
90	330	V3	334	C3	0.43	0
91	413	V4	415	V4	0.43	1
92	232	C2	268	C2	0.43	1
93	658	Ectodomain	662	Ectodomain	0.43	
94	360	C3	362	C3	0.42	1
95	290	C2	344	C3	0.42	1
96	402	V4	403	V4	0.42	
97	149	V1	151	V1	0.42	1
98	4	SP	7	SP	0.42	
99	394	V4	396	V4	0.42	1
100	410	V4	411	V4	0.42	1
101	659	Ectodomain	662	Ectodomain	0.41	
102	462	V5	463	V5	0.41	1
103	360	C3	467	V5	0.41	1
104	22	SP	23	SP	0.41	
105	769	Endodomain	770	Endodomain	0.41	
106	153	V1	154	V1	0.41	1
107	330	V3	332	C3	0.41	1
108	230	C2	240	C2	0.40	1
109	335	C3	412	V4	0.40	1
110	277	C2	352	C3	0.40	1
111	293	C2	446	C4	0.40	1
112	389	V4	417	V4	0.40	1
113	354	C3	357	C3	0.40	1
114	640	Ectodomain	641	Ectodomain	0.40	
115	92	C1	238	C2	0.39	1
116	655	Ectodomain	658	Ectodomain	0.39	
117	674	Ectodomain	677	Ectodomain	0.39	
118	641	Ectodomain	644	Ectodomain	0.39	
119	460	C4	462	V5	0.39	1
120	150	V1	152	V1	0.39	1
121	398	V4	401	V4	0.39	
122	187	V2	190	V2	0.39	
123	396	V4	397	V4	0.39	1
124	358	C3	360	C3	0.39	1
125	149	V1	150	V1	0.39	1
126	464	V5	465	V5	0.39	1
127	320	V3	321	V3	0.38	1
128	148	V1	150	V1	0.38	
129	300	V3	442	C4	0.38	1
130	289	C2	344	C3	0.38	1
131	815	Endodomain	818	Endodomain	0.38	
132	130	C1	132	V1	0.38	1
133	396	V4	400	V4	0.38	0

Continued on next page

Table S1 – continued from previous page

	i (HXB2)	i (Domain)	j (HXB2)	j (Domain)	GREMLIN	TP
134	817	Endodomain	818	Endodomain	0.37	
135	700	TM	758	Endodomain	0.37	
136	442	C4	444	C4	0.37	1
137	32	SP	33	C1	0.37	1
138	357	C3	358	C3	0.37	1
139	339	C3	341	C3	0.37	1
140	273	C2	481	C5	0.37	1
141	192	V2	426	C4	0.37	0
142	401	V4	404	V4	0.37	
143	746	Endodomain	758	Endodomain	0.36	
144	724	Endodomain	726	Endodomain	0.36	
145	671	Ectodomain	683	Ectodomain	0.36	
146	152	V1	153	V1	0.36	1
147	236	C2	275	C2	0.36	1
148	275	C2	282	C2	0.36	1
149	845	Endodomain	851	Endodomain	0.36	
150	151	V1	152	V1	0.36	1
151	837	Endodomain	841	Endodomain	0.36	
152	841	Endodomain	842	Endodomain	0.36	
153	24	SP	25	SP	0.36	
154	154	V1	300	V3	0.35	1
155	665	Ectodomain	667	Ectodomain	0.35	
156	308	V3	316	V3	0.35	1
157	667	Ectodomain	674	Ectodomain	0.35	
158	798	Endodomain	801	Endodomain	0.35	
159	824	Endodomain	825	Endodomain	0.35	
160	817	Endodomain	821	Endodomain	0.35	
161	146	V1	148	V1	0.35	
162	833	Endodomain	837	Endodomain	0.35	
163	816	Endodomain	818	Endodomain	0.35	
164	279	C2	474	C5	0.35	0
165	630	Ectodomain	633	Ectodomain	0.35	
166	178	V2	195	V2	0.35	0
167	840	Endodomain	845	Endodomain	0.34	
168	392	V4	394	V4	0.34	1
169	332	C3	336	C3	0.34	0
170	137	V1	140	V1	0.34	0
171	5	SP	6	SP	0.34	
172	319	V3	322	V3	0.34	1
173	346	C3	393	V4	0.34	1
174	354	C3	356	C3	0.34	1
175	135	V1	138	V1	0.34	1
176	140	V1	141	V1	0.34	
177	147	V1	148	V1	0.34	
178	144	V1	145	V1	0.34	
179	150	V1	154	V1	0.34	0
180	632	Ectodomain	640	Ectodomain	0.34	
181	146	V1	147	V1	0.34	
182	394	V4	395	V4	0.33	1
183	309	V3	315	V3	0.33	1
184	500	C5	619	Ectodomain	0.33	

Continued on next page

Table S1 – continued from previous page

	i (HXB2)	i (Domain)	j (HXB2)	j (Domain)	GREMLIN	TP
185	178	V2	194	V2	0.33	1
186	363	C3	364	C3	0.33	1
187	514	FP	515	FP	0.33	
188	152	V1	155	V1	0.33	1
189	136	V1	139	V1	0.33	1
190	683	Ectodomain	684	Ectodomain	0.32	
191	62	C1	63	C1	0.32	1
192	31	SP	32	SP	0.32	1
193	662	Ectodomain	665	Ectodomain	0.32	
194	444	C4	446	C4	0.32	1
195	388	V4	389	V4	0.32	1
196	25	SP	26	SP	0.32	
197	185	V2	186	V2	0.32	0
198	723	Endodomain	724	Endodomain	0.32	
199	328	V3	330	V3	0.32	1
200	166	V2	167	V2	0.32	1
201	62	C1	65	C1	0.32	1
202	746	Endodomain	747	Endodomain	0.32	
203	295	C2	446	C4	0.32	1
204	269	C2	348	C3	0.31	1
205	21	SP	23	SP	0.31	
206	211	C2	379	C3	0.31	1
207	588	Ectodomain	646	Ectodomain	0.31	
208	10	SP	12	SP	0.31	
209	134	V1	154	V1	0.31	1
210	460	C4	461	V5	0.31	1
211	347	C3	350	C3	0.31	1
212	396	V4	399	V4	0.31	1
213	515	FP	518	FP	0.31	
214	364	C3	365	C3	0.31	1
215	397	V4	400	V4	0.31	0
216	266	C2	269	C2	0.31	1
217	49	C1	99	C1	0.31	1
218	677	Ectodomain	683	Ectodomain	0.31	
219	7	SP	9	SP	0.31	
220	179	V2	192	V2	0.31	0
221	722	Endodomain	723	Endodomain	0.30	
222	289	C2	290	C2	0.30	1
223	337	C3	340	C3	0.30	1
224	667	Ectodomain	668	Ectodomain	0.30	
225	410	V4	413	V4	0.30	1
226	429	C4	432	C4	0.30	1
227	424	C4	425	C4	0.30	1
228	463	V5	465	V5	0.30	1
229	346	C3	395	V4	0.30	1
230	301	V3	303	V3	0.30	1
231	172	V2	305	V3	0.30	1
232	754	Endodomain	756	Endodomain	0.30	
233	535	Ectodomain	536	Ectodomain	0.30	
234	665	Ectodomain	677	Ectodomain	0.30	
235	291	C2	293	C2	0.30	1

Continued on next page

Table S1 – continued from previous page

	i (HXB2)	i (Domain)	j (HXB2)	j (Domain)	GREMLIN	TP
236	851	Endodomain	854	Endodomain	0.30	
237	676	Ectodomain	677	Ectodomain	0.30	
238	47	C1	49	C1	0.30	1
239	30	SP	31	SP	0.30	
240	308	V3	315	V3	0.30	1
241	620	Ectodomain	621	Ectodomain	0.30	
242	161	V2	172	V2	0.29	1
243	167	V2	192	V2	0.29	0
244	619	Ectodomain	621	Ectodomain	0.29	
245	114	C1	202	C2	0.29	0
246	2	SP	4	SP	0.29	
247	722	Endodomain	724	Endodomain	0.29	
248	813	Endodomain	817	Endodomain	0.29	
249	166	V2	169	V2	0.29	0
250	150	V1	151	V1	0.29	1
251	232	C2	234	C2	0.29	1
252	270	C2	346	C3	0.29	0
253	141	V1	142	V1	0.29	
254	583	Ectodomain	588	Ectodomain	0.29	
255	770	Endodomain	773	Endodomain	0.29	
256	719	Endodomain	721	Endodomain	0.29	
257	175	V2	177	V2	0.29	1
258	309	V3	317	V3	0.29	1
259	133	V1	152	V1	0.29	1
260	676	Ectodomain	683	Ectodomain	0.29	
261	461	V5	462	V5	0.29	1
262	671	Ectodomain	677	Ectodomain	0.29	
263	502	C5	507	C5	0.29	
264	816	Endodomain	824	Endodomain	0.29	
265	287	C2	291	C2	0.29	0
266	336	C3	337	C3	0.29	1
267	471	V5	474	C5	0.29	0
268	731	Endodomain	732	Endodomain	0.29	
269	20	SP	21	SP	0.29	
270	149	V1	152	V1	0.29	1
271	308	V3	309	V3	0.28	1
272	399	V4	402	V4	0.28	
273	841	Endodomain	856	Endodomain	0.28	
274	536	Ectodomain	539	Ectodomain	0.28	
275	303	V3	306	V3	0.28	0
276	717	Endodomain	721	Endodomain	0.28	
277	730	Endodomain	732	Endodomain	0.28	
278	145	V1	146	V1	0.28	
279	306	V3	322	V3	0.28	0
280	853	Endodomain	856	Endodomain	0.28	
281	330	V3	415	V4	0.28	1
282	135	V1	140	V1	0.28	0
283	721	Endodomain	723	Endodomain	0.28	
284	750	Endodomain	758	Endodomain	0.28	
285	165	V2	192	V2	0.28	0
286	421	C4	424	C4	0.27	1

Continued on next page

Table S1 – continued from previous page

	i (HXB2)	i (Domain)	j (HXB2)	j (Domain)	GREMLIN	TP
287	281	C2	283	C2	0.27	1
288	395	V4	396	V4	0.27	1
289	731	Endodomain	736	Endodomain	0.27	
290	335	C3	339	C3	0.27	1
291	19	SP	29	SP	0.27	
292	146	V1	154	V1	0.27	
293	31	SP	33	C1	0.27	1
294	10	SP	21	SP	0.27	
295	640	Ectodomain	644	Ectodomain	0.27	
296	832	Endodomain	833	Endodomain	0.27	
297	133	V1	155	V1	0.27	1
298	293	C2	297	V3	0.27	0
299	750	Endodomain	754	Endodomain	0.27	
300	719	Endodomain	720	Endodomain	0.27	
301	12	SP	21	SP	0.27	
302	85	C1	230	C2	0.27	0
303	770	Endodomain	774	Endodomain	0.27	
304	306	V3	321	V3	0.26	1
305	290	C2	291	C2	0.26	1
306	291	C2	344	C3	0.26	0
307	230	C2	234	C2	0.26	1
308	150	V1	153	V1	0.26	1
309	161	V2	192	V2	0.26	0
310	10	SP	20	SP	0.26	
311	289	C2	293	C2	0.26	0
312	232	C2	236	C2	0.26	0
313	343	C3	346	C3	0.26	1
314	340	C3	343	C3	0.26	1
315	137	V1	142	V1	0.26	
316	837	Endodomain	840	Endodomain	0.26	
317	84	C1	578	Ectodomain	0.26	
318	722	Endodomain	726	Endodomain	0.26	
319	343	C3	347	C3	0.26	1
320	825	Endodomain	829	Endodomain	0.26	
321	19	SP	20	SP	0.26	
322	618	Ectodomain	621	Ectodomain	0.26	
323	619	Ectodomain	624	Ectodomain	0.26	
324	668	Ectodomain	677	Ectodomain	0.26	
325	134	V1	137	V1	0.26	1
326	169	V2	170	V2	0.26	1
327	350	C3	351	C3	0.26	1
328	667	Ectodomain	671	Ectodomain	0.26	
329	8	SP	9	SP	0.26	
330	167	V2	426	C4	0.26	0
331	335	C3	336	C3	0.26	1
332	65	C1	68	C1	0.26	1
333	146	V1	150	V1	0.25	
334	393	V4	394	V4	0.25	1
335	172	V2	173	V2	0.25	1
336	565	Ectodomain	646	Ectodomain	0.25	
337	369	C3	373	C3	0.25	1

Continued on next page

Table S1 – continued from previous page

	i (HXB2)	i (Domain)	j (HXB2)	j (Domain)	GREMLIN	TP
338	178	V2	183	V2	0.25	0
339	151	V1	153	V1	0.25	1
340	770	Endodomain	783	Endodomain	0.25	
341	335	C3	337	C3	0.25	1
342	651	Ectodomain	658	Ectodomain	0.25	
343	146	V1	149	V1	0.25	
344	10	SP	23	SP	0.25	
345	236	C2	238	C2	0.25	1
346	232	C2	269	C2	0.25	1
347	346	C3	350	C3	0.25	1
348	287	C2	289	C2	0.25	1
349	65	C1	379	C3	0.25	0
350	347	C3	351	C3	0.25	1
351	788	Endodomain	792	Endodomain	0.25	
352	316	V3	319	V3	0.25	1
353	151	V1	154	V1	0.25	1
354	194	V2	195	V2	0.25	1
355	148	V1	149	V1	0.25	
356	232	C2	240	C2	0.25	0
357	426	C4	429	C4	0.25	1
358	159	V2	174	V2	0.25	1
359	302	V3	306	V3	0.25	0
360	845	Endodomain	854	Endodomain	0.25	
361	825	Endodomain	833	Endodomain	0.25	
362	336	C3	340	C3	0.25	1
363	836	Endodomain	839	Endodomain	0.25	
364	307	V3	316	V3	0.25	1
365	275	C2	474	C5	0.25	0
366	619	Ectodomain	620	Ectodomain	0.25	
367	154	V1	155	V1	0.25	1
368	371	C3	375	C3	0.25	1
369	0		21	SP	0.25	
370	87	C1	240	C2	0.25	0
371	277	C2	278	C2	0.25	1
372	164	V2	165	V2	0.25	1
373	801	Endodomain	825	Endodomain	0.25	
374	9	SP	20	SP	0.25	
375	304	V3	322	V3	0.24	1
376	121	C1	429	C4	0.24	1
377	28	SP	31	SP	0.24	
378	617	Ectodomain	626	Ectodomain	0.24	
379	21	SP	22	SP	0.24	
380	830	Endodomain	833	Endodomain	0.24	
381	325	V3	328	V3	0.24	1
382	797	Endodomain	800	Endodomain	0.24	
383	754	Endodomain	758	Endodomain	0.24	
384	275	C2	281	C2	0.24	0
385	149	V1	154	V1	0.24	0
386	425	C4	432	C4	0.24	1
387	158	V2	173	V2	0.24	1
388	12	SP	23	SP	0.24	

Continued on next page

Table S1 – continued from previous page

	i (HXB2)	i (Domain)	j (HXB2)	j (Domain)	GREMLIN	TP
389	373	C3	375	C3	0.24	1
390	393	V4	396	V4	0.24	1
391	303	V3	323	V3	0.24	1
392	700	TM	706	Endodomain	0.24	
393	749	Endodomain	758	Endodomain	0.24	
394	460	C4	463	V5	0.24	1
395	12	SP	20	SP	0.24	
396	829	Endodomain	836	Endodomain	0.24	
397	173	V2	305	V3	0.24	1
398	805	Endodomain	809	Endodomain	0.24	
399	283	C2	471	V5	0.24	1
400	183	V2	184	V2	0.24	1
401	747	Endodomain	755	Endodomain	0.24	
402	287	C2	481	C5	0.24	1
403	306	V3	323	V3	0.24	0
404	301	V3	302	V3	0.24	1
405	172	V2	198	C2	0.24	0
406	340	C3	344	C3	0.24	1
407	535	Ectodomain	539	Ectodomain	0.24	
408	4	SP	0		0.24	
409	515	FP	519	FP	0.24	
410	674	Ectodomain	676	Ectodomain	0.24	
411	28	SP	30	SP	0.24	
412	306	V3	316	V3	0.24	1
413	234	C2	275	C2	0.24	0
414	303	V3	440	C4	0.24	1
415	302	V3	303	V3	0.23	1
416	238	C2	630	Ectodomain	0.23	
417	287	C2	290	C2	0.23	0
418	335	C3	389	V4	0.23	1
419	270	C2	277	C2	0.23	0
420	202	C2	432	C4	0.23	1
421	781	Endodomain	796	Endodomain	0.23	
422	9	SP	10	SP	0.23	
423	648	Ectodomain	655	Ectodomain	0.23	
424	12	SP	30	SP	0.23	
