# Supplementary material for: Coevolution Analysis of HIV-1 Envelope Glycoprotein Complex
Source: PLoS One. 2015 Nov 18;10(11):e0143245. doi: 10.1371/journal.pone.0143245 (PMC4651434; doi:10.1371/journal.pone.0143245)
Supplement: S2 Table — (PDF) [file pone.0143245.s003.pdf]

Supplementary information for:

Coevolution Analysis of HIV-1 Envelope Glycoprotein Complex

Reda Rawi[1,*], Khalid Kunji[1], Abdelali Haoudi[2,3], Halima Bensmail[1]

1 Computational Sciences and Engineering Center, Qatar Computing Research Institute, Doha, Qatar
2 Division of Genetics and Genomics, Boston Children's Hospital, Harvard Medical School, Boston, MA, USA
3 King Abdullah International Medical Research Center, King Abdulaziz Medical City, Riyadh, Saudi Arabia

∗ Corresponding author
E-mail: rrawi@qf.org.qa (RR)

Table S2: Coevolving residues within V3.

	i (HXB2)	j (HXB2)	GREMLIN	TP
47	321	322	0.55	1
62	306	308	0.51	1
127	320	321	0.38	1
156	308	316	0.35	1
172	319	322	0.34	1
183	309	315	0.33	1
199	328	330	0.32	1
230	301	303	0.30	1
240	308	315	0.30	1
258	309	317	0.29	1
271	308	309	0.28	1
275	303	306	0.28	0
279	306	322	0.28	0
304	306	321	0.26	1
352	316	319	0.25	1
359	302	306	0.25	0
364	307	316	0.25	1
375	304	322	0.24	1
381	325	328	0.24	1
391	303	323	0.24	1
403	306	323	0.24	0
404	301	302	0.24	1
412	306	316	0.24	1
415	302	303	0.23	1
