# Supplementary material for: Coevolution Analysis of HIV-1 Envelope Glycoprotein Complex
Source: PLoS One. 2015 Nov 18;10(11):e0143245. doi: 10.1371/journal.pone.0143245 (PMC4651434; doi:10.1371/journal.pone.0143245)
Supplement: S3 Table — (PDF) [file pone.0143245.s004.pdf]

Supplementary information for:

Coevolution Analysis of HIV-1 Envelope Glycoprotein Complex

Reda Rawi[1,*], Khalid Kunji[1], Abdelali Haoudi[2,3], Halima Bensmail[1]

1 Computational Sciences and Engineering Center, Qatar Computing Research Institute, Doha, Qatar

2 Division of Genetics and Genomics, Boston Children's Hospital, Harvard Medical School, Boston, MA, USA

3 King Abdullah International Medical Research Center, King Abdulaziz Medical City, Riyadh, Saudi Arabia

∗ Corresponding author
E-mail: rrawi@qf.org.qa (RR)

Table S3: Coevolving pairs between residues in V3 and other structural regions in 361 the HIV-1 Env.

	i (HXB2)	i (Domain)	j (HXB2)	j (Domain)	GREMLIN	TP
15	295	C2	297	V3	0.78	1
79	325	V3	419	C4	0.45	1
90	330	V3	334	C3	0.43	0
107	330	V3	332	C3	0.41	1
129	300	V3	442	C4	0.38	1
154	154	V1	300	V3	0.35	1
231	172	V2	305	V3	0.30	1
281	330	V3	415	V4	0.28	1
298	293	C2	297	V3	0.27	0
397	173	V2	305	V3	0.24	1
414	303	V3	440	C4	0.24	1
